# Proteomic Investigation on Grp94-IgG Complexes Circulating in Plasma of Type 1 Diabetic Subjects

**DOI:** 10.1155/2015/815839

**Published:** 2015-06-08

**Authors:** Antonella Roveri, Mattia Zaccarin, Andrea Pagetta, Elisa Tramentozzi, Paola Finotti

**Affiliations:** ^1^Department of Molecular Medicine, Section of Biological Chemistry, University of Padua, Via G. Colombo 3, 35131 Padua, Italy; ^2^Department of Pharmaceutical and Pharmacological Sciences, University of Padua, Largo E. Meneghetti 2, 35131 Padua, Italy

## Abstract

The glucose-regulated protein94 (Grp94) has been found in complexes with IgG in plasma of Type 1 (T1) diabetic subjects; however, the pathogenetic meaning of Grp94-IgG complexes has not yet been elucidated. To shed light on the nature and structure of these complexes *in vivo*, we conducted a proteomic analysis on plasma of both T1 diabetic subjects and healthy control subjects. IgG purified from plasma was submitted to 2D PAGE followed by Western blotting and mass analysis. Grp94 was detected in plasma of all diabetic but not control subjects and found linked with its N-terminus to the IgG heavy chain. Mass analysis of heavy chain of IgG that binds Grp94 also *in vitro*, forming stable complexes with characteristics similar to those of native ones, permitted identifying CH2 and CH3 regions as those involved in binding Grp94. At the electron microscopy, IgG from diabetic plasma appeared as fibrils of various lengthes and dimensions, suggestive of elevated aggregating tendency conferred to IgG by Grp94. The nonimmune nature of complexes turned out to be responsible for the particular stability and structure adopted by complexes in plasma of diabetic subjects. Results are of relevance to understanding the pathogenetic mechanisms underlying diabetes and its complications.

## 1. Introduction

Previous studies indicated that plasma of T1 diabetic subjects contains elevated concentrations of the heat shock protein (HSP) glucose-regulated protein94 (Grp94) [1, 2]. Besides the chaperone function shared with other HSPs, Grp94 displays unique immune-regulatory activity by loading antigens onto molecules of the MHC system for presentation to immune competent cells [3, 4]. Since HSPs are obligatory intracellular, extracellular Grp94 always represents an immunological danger [5, 6] and is an index of altered cell membrane permeability that precedes and accompanies diverse inflammatory and immunological processes [7].

In plasma of T1 diabetic patients Grp94 does not circulate as free protein but always binds to IgG, forming stable complexes that have been interpreted as immune in nature [1, 2]. This was also supported by the finding that anti-Grp94 antibodies are present in diabetic plasma in response to the immune stimulation driven by extracellular Grp94 [2]. Other observations indicated that Grp94-IgG complexes in diabetic plasma can induce an intense activation of inflammatory cell signaling pathways with angiogenic-like transformation of vascular cells [8]. This has led to the proposal that Grp94-IgG complexes might be a marker of increased risk of vascular complications in T1 diabetes [2]. However, studies showing that Grp94 is able to form stable complexes with IgG also* in vitro* and that these complexes partly mimic the effects provoked by native complexes [9] raised the possibility that also* in vivo* Grp94 might form nonimmune complexes with IgG. If actually proved, the nonimmune nature of Grp94-IgG complexes could shed new light on the pathogenetic mechanisms associated with diabetes and its complications.

To address the specific issue of the nature of Grp94-IgG complexes and also to define the structure that complexes might adopt* in vivo*, we conducted an in-depth proteomic analysis and microscopic investigation on IgG purified from plasma of both diabetic and healthy control subjects. Our results let elucidate how the nonimmune binding engaged by Grp94 with IgG* in vivo* is responsible for the formation of complexes that for the particular stability and long persistence in circulation might drive adverse biological effects.

## 2. Materials and Methods

### 2.1. Participants in the Study

The study protocol was approved by Local Ethics Committee and both patients and healthy subjects gave their informed written consent for having blood sample (15 mL) taken in fasting conditions. Ten (5 males and 5 females) age-matched, fasting T1 diabetic patients were recruited among those attending the Center of Metabolic Diseases SOC (Hospital of Rovigo) during their routine visits for checking diabetes status. The mean age (±SD) of diabetic patients was 30.5 (±9.57) yr, their age at onset of diabetes 16.5 (±7.41) yr, and diabetes duration 13.9 (±7.46) yr. The patient whose plasma was also analyzed individually was a 26-year-old male who developed diabetes at the age of 11 yr. Eight (4 males and 4 females) nonsmokers volunteers with mean age (±SD) of 35.4 (±8.2) yr served as control.

### 2.2. IgG Purification from Plasma

Plasma was obtained after centrifugation of individual blood sample and dialyzed. An equal content of proteins of any plasma sample (0.9 mg proteins) served to form the pool of plasmas, and 1.0 mg proteins of pooled plasma were loaded onto a mono-Q HR 5/5 column (Amersham Biosciences, Uppsala, Sweden), as described in [10]. IgG eluted in the first two peaks that were then collected and loaded onto a 1 mL HiTrap Protein G HP column for further purification. The whole IgG eluted at the flow rate of 0.5 mL/min in the second peak with the eluent B (0.1 M glycine buffer, pH 2.5) and acidity was immediately buffered (1.0 M Tris-HCl pH 9.0) to prevent protein denaturation. The IgG peak was ultrafiltered on Amicon Centriplus YM-3 and proteins measured by BCA protein assay. The same procedure was applied to obtain purified IgG from individual diabetic plasma sample.

### 2.3. Analysis on Grp94-IgG Complex Formed* In Vitro*

In separate experiments, recombinant rabbit Grp94 was used to form complexes with human IgG, following the procedure detailed previously [11]. Briefly, Grp94 was incubated with IgG at the molar ratio of 1 : 2, and after incubation at 37°C for 2 h, the complex formation was evaluated in native PAGE by loading 5 *μ*g proteins onto 8.5% polyacrylamide gel, followed by Western blotting for detecting Grp94. The complex solution was then submitted to MS analysis.

### 2.4. Electrophoresis and Western Blot Analyses

SDS-PAGE was performed on 10% polyacrylamide gel and samples (7–10 *μ*g proteins) were loaded either with or without reducing treatment, as specified in the figure legends. Gels were stained with Coomassie Brilliant Blue. For Western blotting, proteins were transferred into a PVDF membrane (Immobilon P, Millipore, Bellerica, MA, USA) and treated with anti-human Grp94 monoclonal (9G10 clone) (Santa Cruz Biotechnology, Santa Cruz, CA, USA) and sheep anti-human whole IgG antibodies (Abs) (Bethyl Laboratories, Inc., Montgomery, TX, USA). For 2D-PAGE, 41.5 *μ*g IgG were diluted to 1.0 mL with 0.1 M Tris-HCl, 1.0 mM EDTA, and 6.0 M guanidine at pH 8.3 and submitted to extensive reduction with DTT and carboxymethylation with iodoacetic acid according to Allen [12] followed by thorough dialysis against water. The IgG solution was then brought to dryness in SpeedVac and dissolved in 5.0 mM Trizma base, 9.0 M urea, 2% (w/v) CHAPS, and 0.2% (w/v) ampholyte, with pH range 3–10. IEF was carried out on 3–10 pH range 11 cm IEF strips for 35,000 V × h at 4°C. Focusing strips were then incubated in 0.375 M Tris-HCl pH 8.8, 2% (w/v) SDS, 6.0 M urea, and 20% (v/v) glycerol for 20 min, immediately loaded at the top of 12% SDS gels, covered with Laemmli reducing sample buffer and run in a stepwise manner at 5 mA/strip for 1 hour, 10 mA/strip for 1 hour, and 20 mA/strip for 3 hours. The SDS gels were either stained with Colloidal Coomassie blue or used for Western blotting.

### 2.5. In-Gel Protein Digestion

Following Western blotting, spots of the proteins of interest were excised from the gel stained with Colloidal Coomassie after SDS-PAGE, and gel pieces destained twice in 0.2 M ammonium bicarbonate, 40% (v/v) acetonitrile at 37°C, and dehydrated with 100% acetonitrile and brought to dryness in SpeedVac. Rehydration of gel pieces was performed on ice in a minimal volume of 40 mM ammonium bicarbonate and 10% (v/v) acetonitrile pH 8.0 containing 0.4 *μ*g trypsin (Trypsin Gold, Mass Spectrometry grade, PROMEGA). Digestion was performed overnight at 37°C. After incubation, peptides were recovered and transferred into a new tube. The sample mixture from each gel piece was thus brought to dryness in SpeedVac and resuspended into a minimal volume of 0.1% (w/v) formic acid for subsequent MS analysis.

### 2.6. MS Analysis

Samples from digested gel spots were run in an LTQ-Orbitrap XL mass spectrometer (Thermo Scientific) coupled with a nano-HPLC Ultimate 3000 (Dionex). Peptides were loaded onto a homemade picofrit column packed with C18 material and separated using a 45 min linear gradient of acetonitrile/0.1% formic acid (from 0% to 40% acetonitrile in 25 min), at a flow rate of 250 nl/min. Capillary voltage was set at around 1.3–1.5 kV and source temperature at 200°C. For identification experiments, the instrument was operating in a data-dependent mode according to a full scan at 60,000 resolution on the Orbitrap, followed by 10 MS/MS scans on the most intense ions in the linear trap.

### 2.7. Analysis of Data

Raw data were visualized and exported into  .xml format with the Protein Identification software BioWorks rev. 3.3.1 SP1 (Thermo Fisher Scientific Inc., Waltham, MA USA) for subsequent database mining. MS/MS dataset was searched with Mascot search engine with in-house server version of Mascot rev. 2.3 (Matrix Science) against* ad hoc* built database for 94 kDa glucose-regulated protein (SwissProt, entry P14625, and O18750) and against the whole SwissProt database with no taxonomy restriction. Search constraints were 10 ppm tolerance for precursor ion masses and 0.6 Da tolerance for fragment ion masses. No enzyme restriction specificity was imposed for peptide, and cysteine residues were assumed to be carboxymethylated in samples from 2D-PAGE, while deamidation on arginine/glutamine, oxidation on methionine, and acrylamide adduct on cysteine were selected as possible modifications.

### 2.8. Electron Microscopy Analysis

Plasma-purified IgG solutions were used at the final concentration of 0.09 mg/mL. An aliquot of each sample was absorbed onto glow-discharged carbon-coated Butvar films on 400-mesh copper grids. The grids were negatively stained with an unbuffered solution of 1% uranyl acetate and observed at the microscope (Tecnai G12, Fei Company, Eindhoven, Holland). For each sample, several pictures were taken in separate sections of the grid and those representative of at least two measurements performed on different occasions were presented.

## 3. Results

### 3.1. Grp94 Is Bound to IgG Heavy Chain of Diabetic Subjects

**Figure 1:**
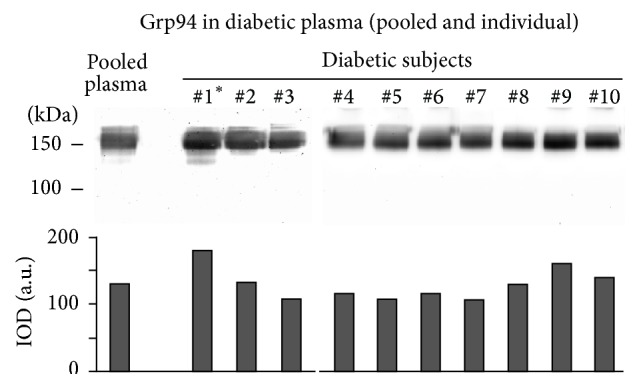
Grp94 is detected in plasma of diabetic subjects. Plasma samples were processed as described in Section 2, and 7.5 *μ*g proteins of each sample loaded onto a 10% polyacrylamide gel without reducing treatment. Pooled plasma was obtained by mixing an equal protein content of any single plasma sample. Proteins were blotted onto a PVDF membrane and probed with anti-Grp94 Abs. Cross-reactivity for secondary anti-human Abs was excluded by exposing the membrane to secondary Abs before the incubation with primary Abs. Densitometric analysis of Grp94-positive bands is reported below (IOD, integrated optical density). ^*∗*^Subject whose plasma was also analyzed separately.

**Figure 2:**
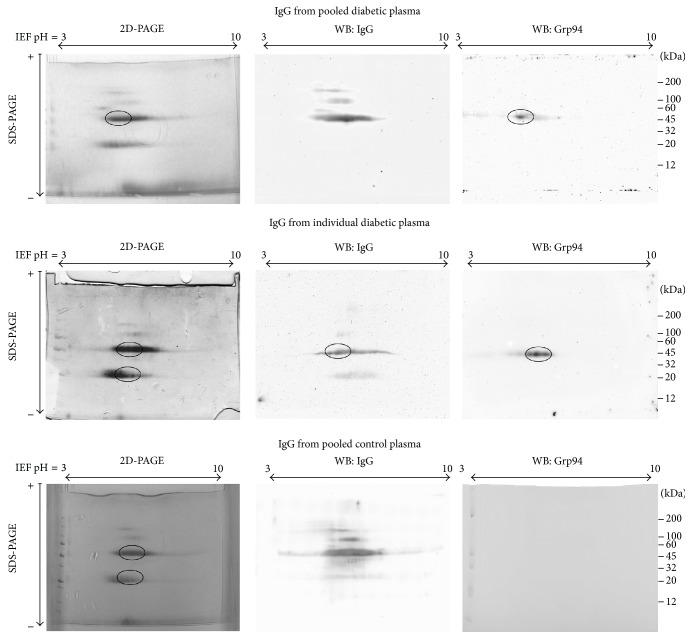
Grp94 is linked to the IgG heavy chain. Panels show 2D SDS-PAGE of the IgG fraction purified from plasma of both diabetic (pooled and single plasma) (upper and central panels, resp.) and healthy control subjects (pooled plasma, lower panels). Protein concentration was measured with the BCA protein assay and 41.5 *μ*g proteins of each sample were loaded in the gel. Correctness of the protein loading was assessed by prior explorative SDS-PAGE. 2D SDS-PAGE of each sample was conducted in duplicate: one gel was used for mass analysis and the other blotted and probed with anti-Grp94 Abs and then with anti-IgG biotin-conjugated Abs followed by exposure to HRP-conjugated streptavidin. After stripping, the membrane was exposed to anti-human IgG primary Abs followed by incubation with HRP-conjugated secondary Abs. Horizontal and vertical arrows indicate, respectively, the range of pH (3–10) at which sample proteins focused in the first iso-focusing PAGE and the direction of the run (from the cathode to the anode) in the second dimension SDS-PAGE. Circled are parts of IgG bands, including those positive for Grp94, excised for mass analysis.

Any individual plasma and pooled plasma of diabetic and control subjects were first analyzed in Western blotting to assess positivity for Grp94. No immune reaction for Grp94 was shown in control plasma (data not shown), whereas an intense positivity was detected in any diabetic plasma, although with some interindividual difference (Figure 1). The high molecular mass at which Grp94 focused in nonreducing conditions of SDS-PAGE was consistent with the formation of complexes with IgG, as assessed by copositivity for IgG in the same Grp94-positive bands (data not shown). 2D SDS-PAGE was performed in parallel on the IgG fraction purified from pooled plasma of both diabetic and control subjects (Figure 2) to detect differences in charge density and/or composition of IgG, and to identify the IgG subunit involved in binding Grp94. The same analysis was also conducted on individual plasma that showed the highest concentration of Grp94 (Figure 1, patient #1). IgG of diabetic and control subjects had similar isoelectric focusing and distribution pattern of IgG subunits in terms of molecular masses. Grp94 was not found in any IgG subunits of control subjects, whereas it was detected in the 50 kDa band of IgG of both pooled and individual diabetic plasmas (Figure 2). In pooled plasma, Grp94 focused on a single spot at acidic pH, likely due to overwhelming contribution of more negatively charged IgG of some patient(s) in the pool, whereas in individual plasma Grp94 focused on a wider range of pH with a higher intensity of the immune reaction. The different distribution pattern of IgG-linked Grp94 was consistent with the observed interindividual variability in the quantity and qualitative characteristics of circulating Grp94-IgG complexes [2]. Results proved that part of Grp94 was still strongly bound to the 50 kDa IgG subunit although most part of it was lost during the extensive denaturation of samples for 2D SDS-PAGE.

### 3.2. N-Terminal Region of Grp94 Is Bound to IgG Heavy Chain

**Figure 3:**
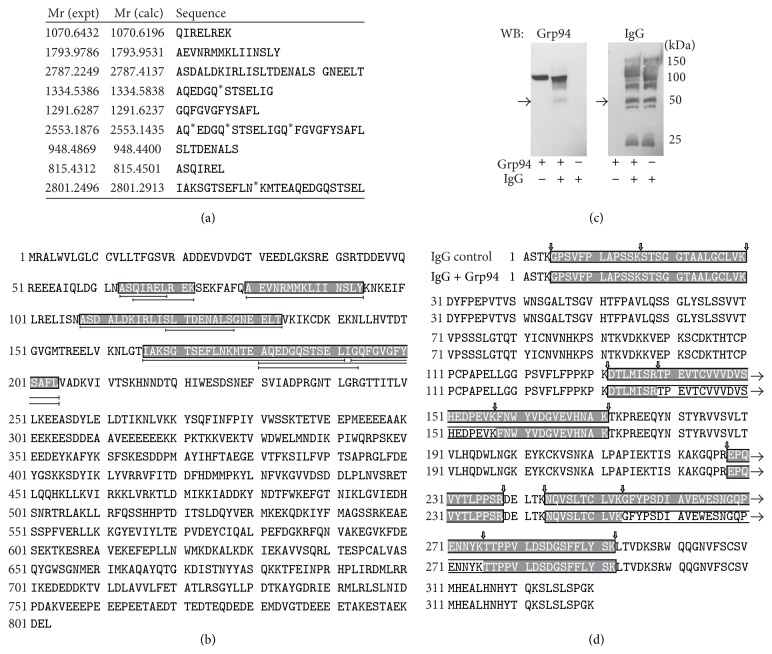
Identification of sites of binding in both Grp94 and IgG. (a) Nine unique peptides mapping the N-terminal region of human Grp94 were obtained after digestion of spots at about 50 kDa in bands positive for both Grp94 and IgG (evidenced in Figure 2) and submitted to ESI-mass analysis. Only one peptide was produced by trypsin digestion, whereas others might be due to autoproteolytic cleavage. Calculated molecular mass of each peptide is reported near the corresponding sequence. ^*∗*^Aminoacid residue deamidated. (b) Sequence of human Grp94 (Hsp90 B1, Swiss Prot entry P14625). Sequences corresponding to the observed peptides are grey boxed. More than one peptide is found to match the boxed sequences (indicated by double arrow lines below the sequence). (c) Western blotting for both Grp94 and IgG of human IgG and recombinant rabbit Grp94 incubated both alone (control) and together at the final molar concentration of 2 : 1 to form Grp94-IgG complexes, as specified in Section 2. Five *μ*g IgG and 1.3 *μ*g Grp94 were loaded onto 10% polyacrylamide gel after reducing treatment of samples. After blotting, the membrane was first exposed to monoclonal anti-Grp94 Abs and then, after stripping, to anti-human IgG Abs. The immune reaction was detected as specified in the legend to Figure 2. The arrow on the left indicates the 50 kDa band excised from the gel of SDS-PAGE for mass analysis and found positive for Grp94. No immune reaction at 50 kDa was found in control Grp94. (d) Human IGHG1 amino acid sequence containing CH1 (residues 1–98), hinge (residues 99–110), CH2 (residues 111–223), and CH3 regions (residues 254–275) in both control IgG and IgG + Grp94. Coverage of the Ig *γ*-1 chain C for both sample was highly reproducible. Peptides identified in both samples are boxed in grey, whereas the missing sequences in IgG + Grp94 are in clear boxes. Empty arrows indicate the cleavage points by trypsin. Arrows on right indicate that the coverage sequence extends to the following residues.

Several spots of IgG bands, of both control and diabetic plasma, including bands positive for Grp94 at 50 kDa, were excised from gels of 2D PAGE and digested for ESI-MS analysis. Analysis showed that bands at both 100 and 50 kDa contained IgG heavy (*γ*) chain and those at 25 kDa light (*k*) chains (data not shown). No sequence pertaining to Grp94 was found in any IgG band of control plasma and also in the 50 kDa IgG band of pooled diabetic plasma although Western blotting proved the presence of Grp94 (Figure 2). This was likely due to the small quantity of Grp94 in the spot, insufficient for being detected in mass analysis. Indeed, in the IgG heavy chain of individual plasma with a more intense positivity for Grp94, mass analysis identified nine unique peptides (Figure 3(a)) comprised between the amino acid residues 63 and 201 in the N-terminal region of human Grp94 (Figure 3(b)). Intriguingly, only one peptide was produced by trypsin cleavage, whereas others were formed by a nonspecific, probably autoproteolytic mechanism. This finding suggested that sites of proteolytic attack in Grp94 were not accessible to trypsin, probably for steric hindrance due to IgG binding.

Mass analysis of Grp94-positive heavy chain did not permit identifying the site in IgG specifically involved in binding Gp94, since tryptic digestion of the heavy chain yielded peptides that always matched those of control IgG heavy chain (data not shown). However, this negative result was somehow expected given the large excess of Grp94-free IgG that rendered any signal due to Grp94-bound IgG undetectable.

### 3.3. CH2 and CH3 Regions of IgG Involved in Binding to Grp94* In Vitro*

To overcome the difficulty to identify the portion of the IgG heavy chain involved in binding Grp94* in vivo*, we took the advantage of the analysis performed on complexes that Grp94 can also form* in vitro* with human IgG [9]. In previous works we demonstrated that these complexes have properties that partly overlap those displayed by native ones, being also characterized by an irreversible binding that confers on these complexes a particular resistance to tryptic digestion [11]. Taking into consideration these results and the finding that in diabetic plasma Grp94 is linked to IgG heavy chain (Figure 2), we hypothesized that also in complexes formed* in vitro* the binding could occur in the same region of IgG. Thus, by investigating* in vitro* formed Grp94-IgG complexes it was possible to make inference about the IgG region involved in Grp94 binding also* in vivo*. To this aim, mass analysis was conducted on heavy chain of human IgG incubated both alone and with recombinant rabbit Grp94, used as relevant substitute of human Grp94 to form nonimmune complexes [11]. Since in these experiments we used stoichiometric quantities of Grp94 and IgG, it was not necessary to submit samples to 2D-PAGE to separate IgG subunits that were easily evidenced by analyzing samples in reducing conditions in SDS-PAGE followed by Western blotting (Figure 3(c)). Thus, by probing samples with both anti-IgG and anti-Grp94 Abs, we noted that the 50 kDa band of IgG incubated with Grp94 was also positive for Grp94 (Figure 3(c)). Since control Grp94 did not migrate at 50 kDa, this result indicated that Grp94 was closely associated with IgG, similarly to what was observed in diabetic plasma (Figure 2). Mass analysis of 50 kDa bands of both control IgG and IgG incubated with Grp94 confirmed the presence of IgG *γ*-1 chain C region in both samples (Figure 3(d), overlapping peptides that are grey boxed). However, in the heavy chain of Grp94-IgG complex, two long peptides: ^139^TPEVTCVVVDVSHEDPEVK in the CH2 region and ^254^GFYPSDIAVEW-ESNGQPENNYK in the CH3 region were missing (Figure 3(d), residues in clear boxes). The lack of coverage of these sequences was consistent with being these regions of IgG involved in binding to Grp94 so that the tryptic cleavage at ^138^R and ^253^K yielded peptides that did not match any known IgG sequences deposited in the data banks.

### 3.4. Electronic Visualization of Grp94-IgG Complexes in Diabetic Plasma

**Figure 4:**
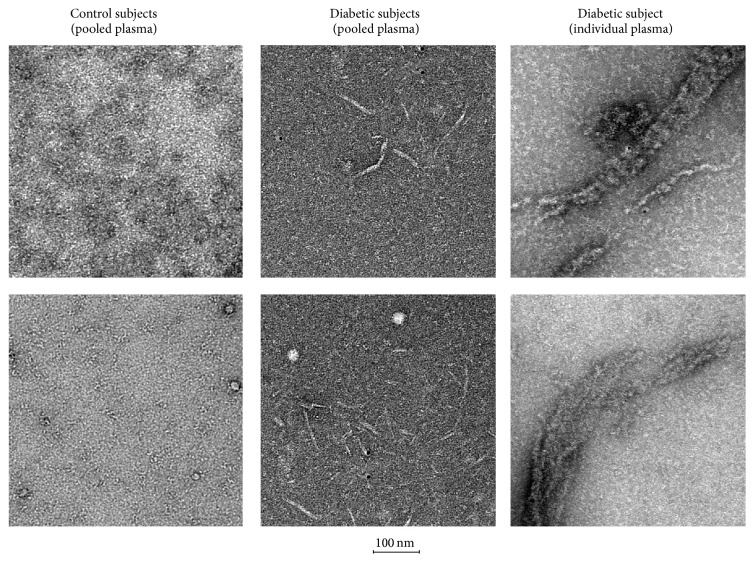
Electron microscopy of IgG purified from both pooled and single diabetic plasma and from pooled control plasma. Two representative images, taken from different fields in the same or separate pictures, are shown for each sample and treated as specified in Section 2. Homogeneous carpet of IgG distributed in overlapping layers with rare aggregates was the common feature of the control sample, whereas filaments of various dimensions were the characteristic shape adopted by IgG in diabetic plasma. Calibration bar is below images.

Since complexes that Grp94 forms with IgG* in vitro* can reach big dimensions [11], we asked whether also complexes circulating in diabetic plasma could be visualized at the electronic microscopy. We thus analyzed IgG of both pooled and individual diabetic plasma and those of control subjects at the electronic microscopy. A dense carpet of IgG molecules, locally aggregated in isolated complexes and characterized by well-known epsilon shape, was the common feature of control plasma, whereas numerous filaments of variable lengths (from few nm up to 100 nm) populated some picture of pooled diabetic plasma (Figure 4). Even bigger and longer filaments (up to 1 *μ*m) were present in individual diabetic plasma in which fibrils looked like amyloid formations. Although differences in the length and dimension appeared between pooled and individual plasma, fibril formations were the hallmark of any diabetic plasma supporting the possibility that the particular aggregating tendency of IgG was the consequence of the complex formation with Grp94.

## 4. Discussion

In this work we wanted to address the unsolved question concerning the nature and structure of Grp94-IgG complexes that circulate in plasma of T1 diabetic subjects with still uncertain pathogenetic significance. Previous observations suggested that these complexes could be immune in nature, based on the finding that anti-Grp94 Abs were present in diabetic plasma in response to extracellular exposure of Grp94 [8]. However, the discovery that Grp94 was able to form rapidly stable complexes also with nonimmune IgG* in vitro* [9] raised the possibility that the mechanism leading to the formation of Grp94-IgG complexes* in vivo* could also exclude an antigenic recognition. In this view, Grp94 that enters circulation following autoimmune cell damage is expected to bind rapidly to nonimmune IgG available in large excess in plasma and form stable complexes that, thanks to their stability, might acquire immunogenic properties [9]. Thus, the complexes that Grp94 forms first might be nonimmune in nature, whereas immune ones appear later in response to the immune stimulation mostly exerted by Grp94 in nonimmune complexes. Our present results support the possibility that Grp94-IgG complexes are actually nonimmune in nature, since mass analysis conducted on IgG purified from diabetic plasma demonstrated that Grp94 was inextricably linked to IgG heavy chain, whereas Grp94 was never found in the IgG light-chain, a result that permitted excluding that the antigen-binding site of IgG is involved in the binding to Grp94. The failure to detect immune Grp94-IgG complexes does not negate the presence of these complexes in the circulation rather it means that only the binding engaged in nonimmune complexes is so strong to survive the drastic procedures to which plasma samples are submitted for mass analysis, whereas reversibility of the antigen-antibody binding does not permit rescuing immune complexes.

Interestingly, we found Grp94 linked to IgG heavy chain in any diabetic plasma, both individual and pooled (Figure 2), although mass analysis identified the peptides of the N-terminus of Grp94 only in plasma of the diabetic subject with the most intense expression of Grp94. The plausible explanation of this result is that, in the pool of plasmas in which any patient gave his/her own variable contribution of Grp94 (Figure 1), the resulting concentration of Grp94 was too low to permit the detection of any Grp94 fragment, considering also the loss of Grp94 due to the drastic conditions used in the mass analysis.

The overwhelming concentration of Grp94-free over Grp94-bound IgG in diabetic plasmas prevented us from identifying the specific portion of the IgG heavy chain engaged in binding to Grp94. However, we circumvented this obstacle by taking advantage of previous observations showing that Grp94 can form complexes with IgG also* in vitro* [9]. The results of mass analysis demonstrating that Grp94 is linked to IgG heavy chain with the N-terminal region (Figure 3(a)) were in line with previous results showing that the N-fragment of Grp94 was* per se* sufficient to establish the binding to IgG* in vitro* [11]. This gave support to the assumption that the reaction of binding occurring in nonimmune Grp94-IgG complexes* in vitro* might be the same that takes place* in vivo*. Thus, complexes formed* in vitro* could be a reliable substitute of native ones for giving information about the region of IgG involved in binding. This approach permitted us to identify in CH2 and CH3 regions of the IgG heavy chain the peptide sequences specifically involved in binding Grp94 (Figure 3(d)) and to extend this result to the IgG heavy chain of native complexes.

The stability and elevated aggregating tendency of circulating Grp94-IgG complexes predicted by results of proteomic analyses were further confirmed by the electron microscopy analysis with the visualization of IgG that in any diabetic, but not control plasma, appeared organized in characteristic fibril formations of various lengthes and dimensions (Figure 4). In individual plasma, fibrils were bigger and longer than those seen in pooled plasma, a difference likely attributable to the higher concentration of Grp94 in individual plasma. However, regardless of the dimension and shape, fibrils were the hallmark of any diabetic plasma, pooled or single, and prove the pathological risk linked to circulating Grp94-IgG complexes. Although we have no direct proof that fibrils actually are the expression of the aggregation capacity acquired by IgG in complexes with Grp94, the alternative possibility that aggregation is due to IgG glycation can reasonably be ruled out since glycation necessary to induce an aggregation state of proteins should have caused alterations in charge density and structure of IgG that we instead failed to detect in both electrophoretic and mass analyses.

In conclusion, our work has permitted to decipher the nature and to show the shape of Grp94-IgG complexes circulating in plasma of T1 diabetic subjects, demonstrating that the exceptional stability and aggregating tendency of these complexes are due to the binding that excludes the immune recognition and involves the N-terminus of Grp94 and CH2 and CH3 regions in IgG heavy chain. Our results convey important information about the role that Grp94-IgG complexes might have in T1 diabetes: the binding mechanism leading to the formation of nonimmune complexes predicts the rapidity at which complexes can form following the extracellular liberation of Grp94 as well as their long persistence in the circulation. The fact that aggregating complexes were found in plasma of diabetic subjects with diabetes lasting for years (see Section 2) testifies that Grp94 might be liberated into the plasma even long after the onset of disease and that Grp94-IgG complexes that thus form drive a risk of vascular complications.
